# Race‐specific prostate cancer outcomes in a cohort of military health care beneficiaries undergoing surgery: 1990–2017

**DOI:** 10.1002/cam4.4787

**Published:** 2022-05-31

**Authors:** Nathan Oehrlein, Samantha A. Streicher, Huai‐Ching Kuo, Avinash Chaurasia, Jacob McFadden, Darryl Nousome, Yongmei Chen, Sean P. Stroup, John Musser, Timothy Brand, Christopher Porter, Inger L. Rosner, Gregory T. Chesnut, Kayla C. Onofaro, Timothy R. Rebbeck, Anthony D’Amico, Grace Lu‐Yao, Jennifer Cullen

**Affiliations:** ^1^ Urology Service, Department of Surgery Walter Reed National Military Medical Center Bethesda Maryland USA; ^2^ Center for Prostate Disease Research, Murtha Cancer Center Research Program, Department of Surgery Uniformed Services University of the Health Sciences Bethesda Maryland USA; ^3^ The Henry M. Jackson Foundation for the Advancement of Military Medicine Bethesda Maryland USA; ^4^ Department of Radiation Oncology Walter Reed National Military Medical Center Bethesda Maryland USA; ^5^ Department of Urology Naval Medical Center San Diego San Diego California USA; ^6^ Tripler Army Medical Center Honolulu Hawaii USA; ^7^ Madigan Army Medical Center Tacoma WA USA; ^8^ Virginia Mason Medical Center Seattle WA USA; ^9^ INOVA Falls Church Virginia USA; ^10^ Division of Population Sciences, Dana Farber Cancer Institute and Department of Epidemiology Harvard TH Chan School of Public Health Boston Massachusetts USA; ^11^ Department of Radiation Oncology, Brigham and Women’s Hospital and Dana Farber Cancer Institute Harvard Medical School Boston Massachusetts USA; ^12^ Sidney Kimmel Cancer Center at Jefferson Philadelphia Pennsylvania USA; ^13^ Jefferson College of Population Health Philadelphia Pennsylvania USA; ^14^ Department of Population and Quantitative Health Sciences Case Western Reserve University Cleveland Ohio USA; ^15^ Case Comprehensive Cancer Center Cleveland Ohio USA; ^16^ Infectious Disease Clinical Research Program Uniformed Services University of the Health Sciences Bethesda Maryland USA; ^17^ Frederick National Laboratory for Cancer Research National Cancer Institute Frederick Maryland USA; ^18^ Eli Lilly and Company Indianapolis Indiana USA; ^19^ Department of Medical Oncology, Sidney Kimmel Cancer Center at Jefferson Sidney Kimmel Medical College Philadelphia Pennsylvania USA

**Keywords:** healthcare disparities, prostatectomy, prostatic neoplasms

## Abstract

**Background:**

There is substantial variability in prostate cancer (PCa) mortality rates across Caucasian American (CA), African American (AA), Asian, and Hispanic men; however, these estimates are unable to disentangle race or ethnicity from confounding factors. The current study explores survival differences in long‐term PCa outcomes between self‐reported AA and CA men, and examines clinicopathologic features across self‐reported CA, AA, Asian, and Hispanic men.

**Methods:**

This retrospective cohort study utilized the Center for Prostate Disease Research (CPDR) Multi‐center National Database from 1990 to 2017. Subjects were consented at military treatment facilities nationwide. AA, CA, Asian, or Hispanic men who underwent radical prostatectomy (RP) for localized PCa within the first year of diagnosis were included in the analyses. Time from RP to biochemical recurrence (BCR), BCR to metastasis, and metastasis to overall death were evaluated using Kaplan–Meier unadjusted estimation curves and adjusted Cox proportional hazards regression.

**Results:**

This study included 7067 men, of whom 5155 (73%) were CA, 1468 (21%) were AA, 237 (3%) were Asian, and 207 (3%) were Hispanic. AA men had a significantly decreased time from RP to BCR compared to CA men (HR = 1.25, 95% CI = 1.06–1.48, *p* = 0.01); however, no difference was observed between AA and CA men for a time from BCR to metastasis (HR = 0.73, 95% CI = 0.39–1.33, *p* = 0.302) and time from metastasis to overall death (HR = 0.67, 95% CI = 0.36–1.26, *p* = 0.213).

**Conclusions:**

In an equal access health care setting, AA men had a shorter survival time from RP to BCR, but comparable survival time from BCR to metastasis and metastasis to overall death.

## Graphical abstract

Previous research has demonstrated significant differences in long‐term prostate cancer outcomes in Caucasian American compared to African American men. This study explored whether racial differences in long‐term oncological outcomes exist among men diagnosed with prostate cancer and treated surgically, in equal access health care settings. While African American men had slightly poorer biochemical recurrence‐free survival compared to Caucasian American men, there were no racial differences observed for distant metastasis‐free survival or overall survival, controlling for detailed clinical factors. In a large racially heterogeneous cohort of men within an equal access health care setting, long‐term prostate cancer outcomes were comparable across race.

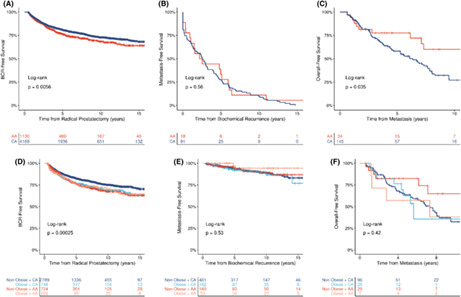

## 1 INTRODUCTION

Prostate cancer (PCa) is the most commonly diagnosed non‐skin malignancy and the fifth leading cause of cancer death in men worldwide, with an estimated incidence of 1.3 million new cases and 359,000 deaths in 2018.^1^ In the United States, population‐based estimates show that African‐American (AA) men are more likely to be diagnosed with PCa, present with distant metastasis, and 2.5 times more likely to die from PCa compared to Caucasian American (CA) men, while Hispanic and Asian men are more likely to be diagnosed with higher stage disease but appear to have comparable oncologic outcomes compared to CA men.^2^ These racial disparities, especially in AA men, are a major health concern and a focus of continual research.^3^

Previous studies that adjusted for relevant confounding variables found a moderate significant or non‐significant decrease in time from radical prostatectomy (RP) to biochemical recurrence (BCR), when comparing AA men to CA men.^4^, ^5^, ^6^, ^7^, ^8^, ^9^, ^10^, ^11^, ^12^, ^13^, ^14^, ^15^, ^16^, ^17^, ^18^, ^19^, ^20^, ^21^, ^22^, ^23^ Fewer studies have examined the association between race and metastasis‐free survival, prostate cancer‐specific mortality, and overall death. These studies report conflicting results: Some suggest that AA men have a moderate decrease in survival time,^24^, ^25^, ^26^, ^27^, ^28^, ^29^, ^30^ whiles others found no difference in survival between AA and CA men. Emerging data from equal access systems and/or adjusting for socioeconomic variables suggest that AA men have non‐inferior oncologic outcomes.^31^, ^32^, ^33^, ^34^, ^35^, ^36^ However, due to the nature of observational study designs, it is difficult to distinguish race from confounding variables such as differences in access to and receipt of healthcare, socioeconomic status, age at diagnosis, and related comorbidities.^37^

In order to further address the relationship between race and long‐term PCa outcomes, a racially diverse, surgically treated cohort of men, enrolled over a 28‐year period in an equal access military health care system was examined. The primary aim of this study was to examine the relationship between CA and AA race and survival time from radical prostatectomy (RP) to BCR, BCR to metastasis, and metastasis to overall survival, controlled for a wide array of clinicopathologic relevant variables. A secondary study aim was to compare clinicopathologic variables across CA, AA, Hispanic, and Asian men.

## 2 METHODS

### 2.1 Study design and participants

A retrospective cohort study was conducted on patients enrolled in the Center for Prostate Disease Research (CPDR) Multi‐Center National Database in men with biopsy‐confirmed PCa who underwent RP treatment within 12 months of diagnosis between January 1, 1990 and December 31, 2017. Enrollees in the CPDR database are military health care beneficiaries who are eligible for TRICARE‐for‐life health care coverage. Demographic, clinical, treatment, and outcomes data were collected as part of routine patient follow‐up on all CPDR enrollees. Informed consent was obtained at the time of transrectal ultrasound‐guided biopsy (TRUS) for suspicion of PCa, as described previously.^38^ Men without self‐reported race, with distant metastasis within a year, local and/or distant metastasis at biopsy, distant metastasis at pathology, or men who underwent neoadjuvant therapy were excluded from the study (Table S1). Institutional Review Board (IRB) approval for data collection and evaluation activities were granted at each participating medical center and the Uniformed Services University of the Health Sciences (USUHS).

### 2.2 Demographic, clinical, and pathologic variables

Patient characteristics of interest in this study included: Age at diagnosis (years), time from RP to last follow up (years), time from diagnosis to RP (months), self‐reported race or ethnicity (CA, AA, Asian, Hispanic), obesity (BMI < 30, BMI ≥ 30), number of medical comorbidities (chronic obstructive pulmonary disease, cardiovascular disease, cerebral vascular accident, and/or cancer), PSA (ng/ml) at time of diagnosis, pathologic stage (T2a, T3‐T4), 2014 ISUP Gleason score (≤6, 3 + 4, 4 + 3, ≥8–10), surgical margin status (positive, negative), primary treatment year (1990–1994, 1995–1999, 2000–2004, 2005–2009, 2010–2014, 2015–2019), post‐BCR PSA doubling time (PSADT) (<3 months, 3–8.9 months, 9.0–14.9 months, >15 months), any secondary treatment, BCR, metastasis, metastasis after BCR, death from any cause, and death from any cause after metastasis.

### 2.3 Study endpoints: Biochemical distant metastasis, and overall death

BCR was defined as a PSA value ≥0.2 ng/ml observed at ≥8 weeks post‐operatively, followed by a subsequent confirmatory PSA level ≥0.2 ng/ml or the initiation of salvage therapy.^39^ Distant metastasis was present if the patient had a positive imaging study in the setting of a rising PSA or a confirmed biopsy result of a distant lesion. A positive imaging study included retroperitoneal lymphadenopathy on magnetic resonance imaging or computerized tomography, or a bone lesion on technetium‐99 m‐HDP bone scan or CT scan.

BCR, distant metastasis, and overall death were modeled as time‐dependent study endpoints with three possible results: Achieved endpoint, censored, or achieved end of study with no event. Men with <6 months of follow‐up time were excluded from each model. As an endpoint, distant metastasis must have occurred more than 1 year after PCa diagnosis. For the BCR endpoint, men were censored at treatment date between RP and BCR, date of last known medical visit, or date of death. For the distant metastasis and death endpoint, men were censored at the date of last known medical visit or date of death. Exclusions were also made for men who underwent adjuvant treatments between RP and BCR, between BCR and metastasis, or between metastasis and death, due to the variability and strong correlation of receipt of treatment with PSADT.

### 2.4 Statistical analysis

All statistical analyses were performed using SAS version 9.4 (SAS Institute, Cary, NC). Frequencies and distributions of patient features were calculated for the study cohort, and stratified by race/ethnicity (CA, AA, Asian, and Hispanic). The chi‐square test and Mann–Whitney *U* test was used to compare categorical and continuous variables, respectively.

A Kaplan Meier curve analysis was used to produce 5‐, 10‐, and 15‐year probability estimates for survival from RP to BCR, BCR to metastasis, and metastasis to overall death as a function of race and race by obesity, restricted to AA and CA men. Cox proportional hazards (PH) analysis was used to model RP to BCR, BCR to distant metastasis, and distant metastasis to overall death as a time‐dependent outcome, as a function of race (CA versus AA). The Cox PH model from RP to BCR was controlled for age at RP, time from diagnosis to RP, primary treatment year, diagnostic PSA, obesity, pathologic stage, Gleason score, surgical margin status, and the number of major comorbidities. The BCR to metastasis Cox PH model was controlled for age at BCR, time from RP to BCR, obesity, pathologic stage, Gleason score, surgical margin status, and PSADT. The metastasis to overall death model was adjusted for age at metastasis, time from RP to metastasis, obesity, and the number of major comorbidities. Hazards ratios (HR) and 95% confidence intervals (CI) were reported for Cox PH models. *P*‐values were computed using two‐sided statistical tests (*α* = 0.05). PH assumptions were checked using the ASSESS PH statement in SAS, and each variable met the PH assumption.^40^

## 3 RESULTS

A total of 7067 men were eligible for the study, of whom 5155 (73%), 1468 (21%), 237 (3%), and 207 (3%) self‐reported as CA, AA, Asian, and Hispanic, respectively (Figure S1 and Table S1). Median patient age at diagnosis and median follow‐up time following RP were 61.6 and 6.7 years, respectively. Among variables that were significantly different across races, CA men were more likely to be older at diagnosis (62.4 years, *p* < 0.001), have a longer time from RP to last follow‐up (7.0 years, *p* < 0.001), have PCa treatment in earlier years (*p* < 0.001), and have a longer post‐BCR PSADT (*p* = 0.11). AA men were less likely to have major comorbidity (87.5%, *p* < 0.001). Hispanic men were more likely to have a lower diagnostic PSA level (5.1 ng/ml, *p* < 0.001). Asian men were less likely to be obese (89.3%, *p* < 0.001), and more likely to have a shorter time from diagnosis to RP (2.0 months, *p* < 0.001), be diagnosed with the lowest pathologic prostate tumor stage and grade (78.4%, T2, *p* = 0.009 and 55.7% ≤ 6, *p* = 0.046, respectively) and to receive another treatment after RP (86.5%, *p* = 0.017) (Table 1). Men with a shorter PSADT also had a shorter time from RP to BCR (*p* < 0.001, Table 2).

**TABLE 1:** Descriptive characteristics of prostate cancer patients overall and by race and ethnicity

Characteristic	All subjects^a^ *N* = 7067	Race					*p*‐value comparing all races
		Caucasian American^a^ *N* = 5155 (73%)	African American^a^ *N* = 1468 (21%)	*p*‐value for CA vs. AA	Asian^a^ *N* = 237 (3%)	Hispanic^a^ *N* = 207 (3%)	
Age at diagnosis (years), median (range)	61.6 (26.7, 84.2)	62.4 (26.7, 84.2)	59 (34.4, 82.9)	<0.001	60.7 (37.9, 77.8)	61.4 (36.5, 77.6)	**<0.001**
Time from RP to last follow‐up (years), median (range)	6.7 (0.5, 28.1)	7.0 (0.5, 28.1)	6.4 (0.5, 26.4)	<0.001	4.9 (0.6, 22.1)	5.8 (0.5, 25.4)	**<0.001**
Time from diagnosis to RP (months), median (range)	2.3 (0.3, 12)	2.2 (0.03, 12)	2.5 (0.03, 12)	<0.001	2.0 (0.6, 11.2)	2.2 (0.5, 11.2)	**<0.001**
Time from RP to BCR (years), median (range) (*N* = 5412)^b^	4.7 (0.5, 22.8)	4.9 (0.5, 22.8)	4.1 (0.5, 21.6)	**<0.001**	3.8 (0.6, 18.4)	4.6 (0.5, 15.6)	**<0.001**
Time from BCR to metastasis (years), median (range) (*N* = 1026)^c^	6.6 (0.5, 23.6)	6.7 (0.5, 22.7)	6.7(0.7, 23.6)	0.96	5.2 (1.3, 18.7)	4.7 (0.6, 18.2)	0.14
Time from metastasis to death (years), median (range) (*N* = 106)^d^	4.1 (0.6, 23.9)	4.1 (0.6, 23.9)	3.9 (0.6, 16.1)	0.88	–	2.6 (2.6, 2.6)	0.84
Obese (*N*, %)	1400 (23.1%)	957 (21.9%)	370 (28.8%)		24 (10.7%)	49 (26.5%)	**<0.001**
Number of comorbidities^e^				<0.001			**<0.001**
0	5645 (79.9%)	3992 (77.4%)	1285 (87.5%)		198 (83.5%)	170 (82.1%)	
1	1226 (17.3%)	996 (19.3%)	161 (11%)		35 (14.8%)	34 (16.4%)	
≥2	196 (2.8%)	167 (3.2%)	22 (1.5%)		4 (1.7%)	3 (1.4%)	
Diagnostic PSA (ng/ml), median (range)	5.5 (0.01, 1060)	5.4 (0.01, 250)	5.8 (0.01, 1060)	<0.001	5.5 (0.1, 76.1)	5.1 (0.04, 27.3)	**<0.001**
Pathologic T stage				0.61			**0.009**
T2	4646 (68.4%)	3366 (67.9%)	963 (68.6%)		182 (78.4%)	135 (68.5%)	
T3–T4	2147 (31.6%)	1594 (32.1%)	441 (31.4%)		50 (21.6%)	62 (31.5%)	
Pathologic Gleason score				0.13			**0.046**
Group 1 (≤6)	3856 (54.6%)	2823 (55.7%)	786 (54.2%)		132 (55.7%)	115 (56.4%)	
Group 2 (3 + 4)	2055 (30.1%)	1507 (29.7%)	420 (28.9%)		67 (28.3%)	61 (29.9%)	
Group 3 (4 + 3)	564 (8.3%)	393 (7.8%)	123 (8.5%)		29 (12.2%)	19 (9.3%)	
Groups 4 & 5 (≥8)	484 (7%)	344 (6.8%)	122 (8.4%)		9 (3.8%)	9 (4.4%)	
Positive surgical margin status	1884 (27.8%)	1363 (27.6%)	389 (27.8%)		74 (32%)	58 (28.9%)	0.52
Year of surgical treatment				<0.001			**<0.001**
1990–1994	1406 (19.9%)	1130 (21.9%)	231 (15.7%)		19 (8%)	26 (12.6%)	
1995–1999	1564 (22.1%)	1134 (22%)	339 (23.1%)		45 (19%)	46 (22.2%)	
2000–2004	1748 (24.7%)	1216 (23.6%)	391 (26.6%)		69 (29.1%)	72 (34.8%)	
2005–2009	1341 (19%)	977 (19%)	263 (17.9%)		58 (24.5%)	43 (20.8%)	
2010–2014	776 (11%)	545 (10.6%)	170 (11.6%)		43 (18.1%)	18 (8.7%)	
2015–2019	232 (3.3%)	153 (3%)	74 (5%)		3 (1.3%)	2 (1%)	
Post‐BCR PSADT^d^ ^,^ ^e^ (*N* = 1020)							
<10 months	193 (18.9%)	135 (18.3%)	52 (22.4%)	0.17	4 (15.4%)	2 (8%)	0.243
> = 10 months	827 (81.1%)	602 (81.7%)	180 (77.6%)		22 (84.6%)	23 (92%)	

Treatment after RP to prevent BCR	239 (4.2%)	181 (4.3%)	47 (4.2%)	0.79	5 (2.7%)	6 (3.7%)	0.78
Treatment after BCR to prevent metastasis	54 (4.8%)	42 (5.1%)	11 (4.3%)	0.60	1 (3.6%)	–	0.79
Treatment after metastasis to prevent death	79 (39.9%)	64 (41%)	13 (34.2%)	0.44	2 (66.7%)	–	0.62
Biochemical recurrence events (*N* = 5651)	1134 (20.1%)	821 (19.7%)	256 (22.7%)		28 (14.8%)	29 (17.8%)	
Distant metastatic events	198 (3%)	156 (3.2%)	38 (2.8%)		3 (1.4%)	1 (0.5%)	–
Distant metastatic events after biochemical recurrence (*N* = 1134)	89 (7.8%)	71 (79.8%)	16 (18.0%)		2 (2.2%)	0	–
All deaths	1028 (15.5%)	824 (16.9%)	169 (12.4%)		19 (8.7%)	16 (8.4%)	–
All deaths after metastasis (*N* = 198)	98 (49.5%)	82 (83.7%)	14 (14.3%)		2 (3.7%)	0	–

*Note*: Abbreviations: AA, African American; BCR, biochemical recurrence; CA, Caucasian American; PSA, prostate‐specific antigen; PSADT, PSA doubling time; RP, radical prostatectomy.

^a^ Number (%) of subjects unless stated otherwise.

^b^ Men who received treatment between RP and BCR were removed from analysis; *N* = 3988 for CA men, *N* = 1083 for AA men, *N* = 184 for Asian men, and *N* = 157 for Hispanic men.

^c^ Men who received treatment between BCR and metastasis were removed from analysis; *N* = 741 for CA men, *N* = 233 for AA men, *N* = 25 for Asian men, *N* = 27 for Hispanic men.

^d^ Men who received treatment between metastasis and death were removed from analysis; *N* = 83 for CA men, *N* = 22 for AA men, *N* = 1 for Hispanic men.

^e^ Comorbidity was defined as chronic obstructive pulmonary disease, cardiovascular disease, cerebral vascular accident, and/or cancer.

**TABLE 2:** Median time between RP and BCR, BCR and metastasis, and metastasis and death in men without treatment to prevent endpoint, overall and by PSA doubling time (*N* = 7067)

Characteristic	All Subjects^a^	PSA doubling time		*p*‐value
		<10 months	≥10 months	
Time from RP to BCR (years), median (range) (*N* = 956)^a^	2.1 (0.5, 17.6)	1.7 (0.5, 12)	2.3 (0.5, 17.6)	<0.001
Time from BCR to metastasis (years), median (range) (*N* = 789)^b^	6.7 (0.5, 23.6)	7.5 (0.5, 22.3)	6.5 (0.5, 23.6)	0.61
Time from to metastasis to death (years), median (range) (*N* = 51)^c^	4.4 (0.6, 23.9)	5.6 (0.6, 23.9)	3.9 (0.6, 13.7)	0.23

^a^ *N* = 185 for PSA doubling time < 10 months and *N* = 772 for PSA doubling time ≥ 10 months.

^b^ *N* = 164 for PSA doubling time < 10 months and *N* = 789 for PSA doubling time ≥ 10 months.

^c^ *N* = 21 for PSA doubling time < 10 months and *N* = 30 for PSA doubling time ≥ 10 months.

**FIGURE 1:**
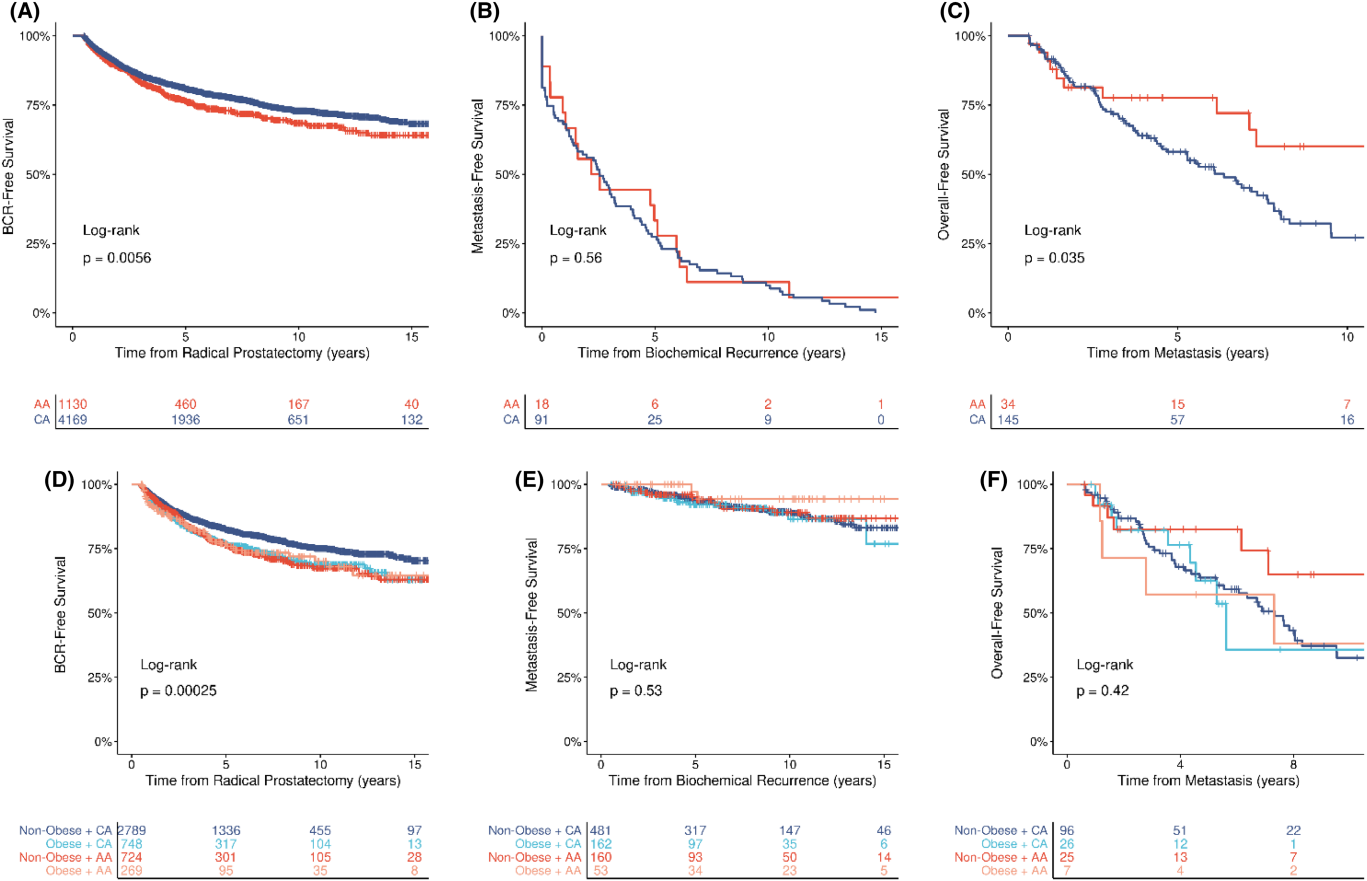
Race‐ and obesity‐stratified Kaplan–Meier estimation curves for 5‐, 10‐, and 15‐year probability estimates of each study outcome. These time to event models were estimated for the following: (A) Radical prostatectomy to biochemical recurrence, (B) Biochemical recurrence to metastasis, (C) Metastasis to all‐cause death, (D) Radical prostatectomy to biochemical recurrence, (E) Biochemical recurrence to metastasis, and (F) Metastasis to overall death, with stratification by race, alone, and race‐by‐obesity subgroups (*n* = 6623*). Abbreviations: AA, African American; CA, Caucasian American; BCR, biochemical recurrence. *Race comparisons were limited to AA versus CA due to sample size constraints for other racial/ethnic groups.

There were 1134 (20%) men who had BCR in the overall cohort: 821 (20%) CA men and 256 (23%) AA men (Table 1). The Kaplan–Meier analysis demonstrated that AA men had a significantly shorter BCR‐free survival time compared to CA men (log‐rank *p* = 0.005) (Figure 1A). Additionally, compared to non‐obese CA men, obese CA men, non‐obese AA men, and obese AA men all had similar and shorter BCR‐free survival times (log‐rank *p* = 0.0002). (Figure 1D).

Of the 1134 men who had BCR, there were 89 (8%) men who had distant PCa metastasis following BCR in the overall cohort: 71 (80%) CA men and 16 (18%) AA men. The Kaplan–Meier analysis demonstrated that there was no difference in survival from BCR to metastasis among CA men compared to AA men (log‐rank *p* = 0.154) (Figure 1A). There was also no difference in survival from BCR to metastasis when evaluated by race and obesity (log‐rank *p* = 0.535) (Figure 1E).

Of the 198 men who had metastatic PCa, there were 98 (49%) men who died following their metastatic PCa in the overall cohort: 82 (84%) CA men and 14 (14%) AA men (Table 1). Kaplan–Meier analysis demonstrated that AA men had a significantly longer survival time between metastasis and overall death compared to CA men (log‐rank *p* = 0.035) (Figure 1A). When evaluated by race and obesity, there was no difference in survival time from metastasis to overall death (log‐rank *p* = 0.421) (Figure 1E).

Survival time from RP to BCR was shorter for AA men compared to CA men (HR = 1.25, 95% CI = 1.06–1.48, Table 3) However, there was no difference in survival time between AA and CA men from BCR to metastasis (HR = 0.73, 95% CI = 0.39–1.33) and from metastasis to overall death (HR = 0.67, 95% CI = 0.36–1.26).

**TABLE 3:** Multivariable Cox proportional hazards models predicting time to event outcomes

Variable	Radical prostatectomy to biochemical recurrence (*N* = 3950)^a^ ^,^ ^b^		Biochemical recurrence to prostate cancer metastasis (*N* = 724)^a^		Prostate cancer metastasis to overall death (*N* = 154)^a^	
	HR (95% CI)	*p*	HR (95% CI)	*p*	HR (95% CI)	*p*
Age at radical prostatectomy (years)	1.01 (0.998, 1.02)	0.101				
Age at Biochemical recurrence (years)			0.99 (0.95, 1.03)	0.513		
Age at metastasis (years)					1.01 (0.93, 1.09)	0.854
Time from diagnosis to radical prostatectomy (years)	1.22 (0.59, 2.51)	0.592				
Time from radical prostatectomy to biochemical recurrence (years)			0.95 (0.83, 1.09)	0.444		
Time from radical prostatectomy to metastasis (years)					1.12 (0.98, 1.29)	0.101
Primary treatment year (1990–2017)	1.01 (0.997, 1.03)	0.114				
Prostate‐specific antigen level at diagnosis (ng/ml)	1.01 (1.00, 1.02)	**0.040**				
Race						
AA vs. CA	1.05 (0.88, 1.25)	0.595	0.75 (0.41, 1.38)	0.363	0.68 (0.24, 1.95)	0.476
Obesity						
Yes vs. No	1.15 (0.97, 1.37)	0.118	0.84 (0.46, 1.51)	0.550	0.53 (0.19, 1.50)	0.233
Pathologic Gleason score						
3 + 4 vs. 3 + 3	0.94 (0.78, 1.12)	0.476	1.44 (0.77, 2.67)	0.249		
4 + 3 vs. 3 + 3	1.14 (0.87, 1.49)	0.342	0.83 (0.33, 2.08)	0.688		
8 ~ 10 vs. 3 + 3	1.25 (0.98, 1.43)	0.079	2.29 (1.18, 4.42)	**0.014**		
Margin status						
Yes vs. No	1.19 (0.98, 1.43)	0.078	0.87 (0.48, 1.58)	0.652		
Pathologic T‐stage						
T3‐T4 vs. T2	0.95 (0.79, 1.16)	0.631	1.39 (0.74, 2.61)	0.306		
Number of comorbidities						
1 vs. 0	1.15 (0.95, 1.40)	0.151			1.07 (0.45, 2.52)	0.880
> = 2 vs. 0	0.98 (0.68, 1.42)	0.923			6.52 (1.04, 41.08)	**0.046**
Prostate‐specific antigen doubling time						
> = 10 vs. <10	0.73 (0.60, 0.89)	**0.002**	0.35 (0.21, 0.60)	**<0.001**	1.07 (0.46, 2.46)	0.876

*Note*: Abbreviations: 95% CI, 95% Confidence Interval; HR, Hazard Ratio.

^a^ Men with <6 months of follow‐up time after radical prostatectomy were removed from the analysis.

^b^ Men who received treatment after radical prostatectomy were censored at treatment.

## 4 DISCUSSION

The relationship between race and survival time from RP to BCR, BCR to metastasis, and metastasis to overall survival (OS), controlled for a wide array of demographic, clinical, and pathological relevant variables was examined as the primary study aim. Comparison of clinicopathologic variables across CA, AA Hispanic, and Asian race or ethnicity was assessed as a secondary study aim. Both analyses were conducted in a large retrospective cohort of racially diverse PCa patients treated with RP and enrolled in an equal access military health care system over a 28‐year period. This setting reduces the disparity in health care access, treatment, and education, which may influence PCa progression and provides a deeper analysis adjusted for potential confounders. This study supports that there is a positive association between AA race and BCR‐free survival when compared to CA race, but no difference between CA and AA race and survival time between BCR and metastasis, and metastasis and overall death. Furthermore, this analysis revealed that Asian and Hispanic American men have similar or better clinical and pathologic PCa features when compared to CA men. To our knowledge, this is the first study to look at long‐term PCa outcomes on a continuum: From RP to BCR, BCR to metastasis, and metastasis to overall survival, one of the largest RP cohorts comparing long‐term PCa outcomes for CA and AA men, and one of the largest known post‐RP cohorts of Asian and Hispanic American men.^41^

Similar to other studies that have examined race and BCR‐free survival, our analysis also revealed a moderate significant decrease in BCR‐free survival time when comparing AA men to CA men, adjusted for confounding variables. When BCR occurs, the treatment team often recommends either androgen‐deprivation (HT) and/or salvage external beam radiation treatment (EBRT) usually happens. In our cohort, there was no significant difference between race and receipt of treatment after BCR (55% AA men vs. 50% CA men, *p* = 0.18). However, despite a shorter BCR‐free survival time for AA men with a near equal proportion of men receiving treatment after BCR, there was no difference in survival time between BCR and metastasis and metastasis and overall death when comparing AA men to CA men. These data are confirmed by a recent analysis on patients who received HT after BCR in an equal access system, showing that race was not a predictor of DM or other adverse outcomes. Additionally, a recent analysis with the entire Department of Defense database revealed no difference in overall survival between AA and CA men diagnosed with PCa.^42^ Together, these findings suggest that AA men may have the less aggressive disease after BCR and/or that AA men may respond better to treatment after BCR conferring improved metastasis‐free survival, prostate cancer‐specific survival, and overall survival for AA men. To this extent, there are a few studies showing that prostate tumors in AA men are more hormonally driven and thus more castrate‐sensitive, possibly leading to better responses to treatment.^43^, ^44^

Freedland and colleagues^13^ were the only other group able to examine AA and CA various long‐term PCa outcomes within one cohort, from the Shared Equal Access Regional Cancer Hospital (SEARCH) database. They found a significant decrease in BCR‐free survival for AA men as compared to CA men on univariable analysis, but not after adjustment for multiple covariates; no difference in aggressive PSA recurrence, metastasis‐free survival, prostate cancer‐specific survival, or overall death for AA men compared to CA men were observed. While their results were comparable to this study's analyses, our data excluded patients who had neoadjuvant therapy, censored all patients who had treatment after RP in the BCR‐free survival models, controlled for comorbidity illness information, and confirmed that treatment after BCR and after metastasis was equal between AA and CA men, ensuring high accuracy of our data, notably in the context of an equal access healthcare setting.

Lee and colleagues^19^ evaluated the interaction between obesity and race on BCR‐free survival and found that BCR‐free survival decreased along the following continuum: Non‐obese CA, obese CA, non‐obese AA, and obese AA; however, this association was not significant following adjusted for pathological tumor stage and grade, and age. Obese CA men, non‐obese AA men, and obese‐AA men all had a similar decreased BCR‐free survival time compared to non‐obese CA men. These findings suggest that while obesity is associated with BCR‐free survival in CA men, it may not be for AA men. This relationship merits further investigation to establish the biological basis of the relationship between obesity, race, and prostate cancer outcomes.

Population studies have found different PCa outcomes among subgroups of both Asian men and Hispanic men.^45^, ^46^, ^47^, ^48^ Previous reports among Asian men have reported higher stages of disease post‐RP and increased likelihood of presentation with advanced stage disease but generally have equal outcomes. Among Hispanic men, it appears that there is no difference in prostate cancer‐specific mortality when comparing to other races, though outcomes may be slightly different when accounting for specific country of origin. Similar clinicopathologic features were found among post surgically treated Asian and Hispanic men when compared to CA men; however, further research needs to be done to examine clinicopathologic features among sub‐groups of Asian and Hispanic PCa patients and to determine long‐term PCa outcomes in Asian and Hispanic men.

There are important limitations to consider in interpreting these findings. First, this study focused only on men who were eligible for RP opted to treat their disease surgically. Therefore, these men are likely younger and healthier than older men or men with multiple comorbidities. While this fact does provide a more homogenous cohort, results may not be applicable to men who choose other treatment modalities, such as definitive radiation. Another study limitation was that lymph node status was not systematically reported for the vast majority of subjects over the 27 year study period and, therefore, could not be examined in multivariable models. Additionally, prostate cancer‐specific mortality was not collected; however, survival time from PCa metastasis to overall death was modeled, which can serve as a surrogate for prostate cancer‐specific mortality. Furthermore, focusing on OS as the outcome may avoid potential biases and inaccuracies inherent in identifying cause‐specific mortality using death certificates.^42^

It is a key strength of the cohort examined in this longitudinal study that all men were enrolled within the military health care system and shared common health care access and insurance providers. All study subjects were military health care beneficiaries with access to TRICARE‐for‐life health care coverage. While we were unable to account for individual‐level education and income levels, this military cohort is fairly homogeneous with respect to SES factors. Also, detailed clinical information was available on study subjects which is a unique attribute to the database from which this study cohort was drawn.

Despite a younger age at diagnosis, increased obesity, higher initial PSA levels, and shorter BCR‐free survival, AA men who underwent RP as primary treatment for PCa had non‐significantly increased survival times between BCR and metastasis and metastasis and OS, compared to CA men in an equal access healthcare system with minimal barriers to healthcare education and access. In concert with the recent results from the numerous studies that have reported only moderate or minimal differences between AA and CA men for long‐term PCa outcomes and highlight the need to decrease racial disparities in access to care.

## AUTHOR CONTRIBUTIONS

Nathan Oehrlein: Conceptualization, investigation, methodology, project administration, resources, supervision, validation, writing ‐ original draft, and writing ‐ review and editing. Samantha A. Streicher: Writing ‐ review and editing. Huai‐Ching Kuo: Data curation, formal analysis, methodology, software, visualization, and writing ‐ review and editing Avinash Chaurasia: Writing ‐ review and editing. Jacob McFadden: Writing ‐ review and editing. Darryl Nousome: Formal analysis, writing ‐ review and editing. Yongmei Chen: Data curation, formal analysis, methodology, software, visualization, and writing ‐ review and editing. Sean P. Stroup: conceptualization, investigation, project administration, validation, and writing ‐ review and editing. John Musser: Conceptualization, investigation, project administration, validation, and writing ‐ review and editing. Timothy Brand: Conceptualization, investigation, project administration, validation, and writing ‐ review and editing. Christopher Porter: Conceptualization, investigation, project administration, validation, and writing ‐ review and editing. Inger L. Rosner: Conceptualization, investigation, project administration, validation, and writing ‐ review and editing. Gregory T. Chesnut: Conceptualization, investigation, project administration, validation, and writing ‐ review and editing. Kayla Onofaro: Investigation, writing ‐ review and editing. Timothy R. Rebbeck: Writing ‐ review and editing. Anthony D'Amico: Conceptualization, Funding acquisition, Writing ‐ original draft, Writing ‐ review and editing. Grace Lu‐Yao: Conceptualization, Funding acquisition, Writing ‐ original draft, Writing ‐ review and editing. Jennifer Cullen: Conceptualization, data curation, funding acquisition, investigation, methodology, project administration, resources, supervision, writing ‐ original draft, and writing ‐ review and editing.

## CONFLICT OF INTEREST

The authors declare no potential conflicts of interest.

## DISCLAIMERS

The contents of this publication are the sole responsibility of the author(s) and do not necessarily reflect the views, opinions or policies of Uniformed Services University of the Health Sciences (USUHS), The Henry M. Jackson Foundation for the Advancement of Military Medicine, Inc., the Department of Defense (DoD), the Departments of the Army, Navy, or Air Force. Mention of trade names, commercial products, or organizations does not imply endorsement by the U.S. Government.

## PRECIS

Among men undergoing radical prostatectomy in an equal access health care setting, we identified and described a cohort of African American, Caucasian, Asian, and Hispanic men. While African American men had an increased risk of biochemical recurrence compared to Caucasian American men after controlling for prostate cancer prognostic factors, African American men experienced a similar risk of metastasis and overall death compared to Caucasian American men.

## Supporting information


Figure S1

## Data Availability

The data supporting the findings of this study are available from the Center for Prostate Disease Research (CPDR). Restrictions and additional conditions may apply to the availability of these data from the Defense Health Agency (DHA).
