# Supplementary material for: Race‐specific prostate cancer outcomes in a cohort of military health care beneficiaries undergoing surgery: 1990–2017
Source: Cancer Med. 2022 May 31;11(22):4354–65. doi: 10.1002/cam4.4787 (PMC9678085; doi:10.1002/cam4.4787)
Supplement: Supplementary file 1 — Figure S1 [file CAM4-11-4354-s001.docx]

**Supplementary Figure 1.** Flow diagram of retrospective study cohort identification process

CPDR^a^ Multi-Center National Database enrollees with biopsy-confirmed prostate cancer who underwent RP^b^ treatment within 12 months of diagnosis between January 1, 1990 and December 31, 2017 (N=7,802)

n=7,802

| **Excluded:** |  |
| --- | --- |
| Patients without self-reported race | (n=126) |
| Patients with distant metastasis within a year | (n=47) |
| Patients with local and/or distant metastasis at biopsy | (n=13) |
| Patients with distant metastasis at pathology | (n=6) |
| Patients who underwent neoadjuvant therapy | (n=543) |

**Final study cohort**

N=7,067

^a^WRNMMC, Walter Reed National Military Medical Center. ^b^RP, radical prostatectomy. ^c^CPDR, Center for Prostate Disease Research. ^d^NCCN, National Comprehensive Cancer Network.
